# Effect of colorectal cancer-derived extracellular vesicles on the immunophenotype and cytokine secretion profile of monocytes and macrophages

**DOI:** 10.1186/s12964-018-0229-y

**Published:** 2018-04-24

**Authors:** Ineta Popēna, Artūrs Ābols, Līga Saulīte, Kārlis Pleiko, Elīna Zandberga, Kaspars Jēkabsons, Edgars Endzeliņš, Alicia Llorente, Aija Linē, Una Riekstiņa

**Affiliations:** 1 Faculty of Medicine, University of Latvia, Raina blvd. 19, Riga, LV-1568 Latvia; 2 Latvian Biomedical Research and Study Centre, Ratsupites iela 1, Riga, LV-1067 Latvia; 3 Department of Molecular Cell Biology, Institute for Cancer Research, Oslo University Hospital-The Norwegian Radium Hospital, 0379 Oslo, Norway

**Keywords:** Extracellular vesicles, cytokines, endocytosis, monocytes, macrophages, colorectal cancer

## Abstract

**Background:**

Macrophages are one of the most important players in the tumor microenvironment. The polarization status of tumor associated macrophages into a pro-inflammatory type M1 or anti-inflammatory type M2 may influence cancer progression and patient survival. Extracellular vesicles (EVs) are membrane-bound vesicles containing different biomolecules that are involved in cell to cell signal transfer. Accumulating evidence suggests that cancer-derived EVs are taken up by macrophages and modulate their phenotype and cytokine profile. However, the interactions of cancer-derived EVs with monocytes and macrophages at various differentiation and polarization states are poorly understood. In the current study, we have analyzed the uptake and functional effects of primary (SW480) and metastatic (SW620) isogenic colorectal cancer (CRC) cell line-derived EVs on monocytes (M), inactive macrophages (M0) and M1 and M2 polarized macrophages.

**Methods:**

THP-1 monocytes were differentiated into M0 macrophages by addition of phorbol-12-myristate-13-acetate. Then M0 macrophages were further polarized into M1 and M2 macrophages in the presence of LPS, IFN- γ, IL-4, and IL-13 respectively. Internalization of SW480 and SW620-derived EVs was analyzed by flow cytometry and fluorescence microscopy. Changes in monocyte and macrophage immunophenotype and secretory profile upon EV exposure were analyzed by flow cytometry, quantitative PCR and Luminex assays.

**Results:**

THP-1 monocytes and M0 macrophages efficiently take up SW480 and SW620-derived EVs, and our results indicate that dynamin-dependent endocytic pathways may be implicated. Interestingly, SW480 and SW620-derived EVs increased CD14 expression in M0 macrophages whereas SW480-derived EVs decreased HLA-DR expression in M1 and M2 polarized macrophages. Moreover, SW480-derived EVs significantly increased CXCL10 expression in monocytes and M0 macrophages. In contrast, SW620-derived EVs induced secretion of IL-6, CXCL10, IL-23 and IL-10 in M0 macrophages. However, addition of CRC cell line-derived EVs together with LPS, IFN- γ (M1) and IL-4, IL-13 (M2) stimuli during macrophage polarization had no additional effect on cytokine expression in M1 and M2 macrophages.

**Conclusion:**

Our results suggest that CRC cell line-derived EVs are internalized and reprogram the immunophenotype and secretory profile in monocytes and inactive macrophages inducing mixed M1 and M2 cytokine response. Although CRC EVs decreased HLA-DR expression in M1, M2 polarized macrophages, their effect on the secretory profile of M1 and M2 polarized macrophages was negligible.

**Electronic supplementary material:**

The online version of this article (10.1186/s12964-018-0229-y) contains supplementary material, which is available to authorized users.

## Background

Extracellular vesicles (EVs) are important mediators of tumor-host interactions [1]. EVs contain several classes of biomolecules such as proteins, lipids and carbohydrates, as well as different kinds of RNA (mRNA, miRNA, piRNA, rRNA, tRNA fragments and other non-coding RNAs). Based on their cargo, EVs are regarded as molecular signal transfer mediators between cells, and are believed to be involved in the regulation of biological processes and to target cell functions and gene expression in recipient cells [2]. For instance, autocrine signals mediated by EVs from non-small cell lung cancer cell lines, glioma cells and gastric cancer cell lines increased cellular proliferation [3, 4]. It was also shown recently that cancer-derived EVs are able to induce angiogenesis, a process inherent to cancer progression [5]. Moreover, cancer-derived EVs actively contribute to metastasis by modulating the microenvironment via degradation of the extracellular matrix and an increased production of pericellular hyaluronic acid, thus facilitating the invasion of cancer cells. Additionally, EVs may contribute to the formation of a metastatic niche in distant tissues via the induction of growth factor signals such as vascular endothelial growth factor, fibroblast growth factor and transforming growth factor beta (TGF-β) [6, 7]. The immunomodulatory effects of tumor derived EVs may reduce the activity of natural killer cells, cytotoxic T cells, and antigen presenting cells [8].

Macrophages are one of the most abundant immune cell types present in the tumour microenvironment (TME) [9]. Two major polarization states have been described for macrophages: the classically activated type (M1) and the alternatively activated type (M2). M1 macrophages are induced by T helper type 1-like cytokines such as IFN-γ and microbial lipopolysaccharides. They produce pro-inflammatory cytokines, chemokines and reactive nitrogen/oxygen intermediates, and thus are involved in antimicrobial and tumoricidal activity. In contrast, M2 macrophages are induced by IL-4 and IL-13 and show immunoregulatory, anti-inflammatory and tumor-promoting activity [10]. The majority of macrophages present in the TME have characteristics of M2 polarized cells, and it is well accepted that tumor associated macrophages (TAMs) have pro-tumorigenic properties [11]. A high number of macrophages in various solid tumors like colorectal cancer (CRC) [12], lung cancer [13], gastric cancer [14] and hormone receptor-positive and triple-negative breast cancer [15, 16] is associated to patients with poor prognosis in terms of overall survival, disease-free survival, metastasis formation and lymph node involvement [17]. TAMs are regarded as pivotal to tumor progression and metastasis, which is the leading cause of cancer-associated mortality [18].

In the present study we have investigated the uptake of primary and metastatic CRC cell line-derived EVs in THP-1 monocytes, and the effect that these EVs have on the immunophenotype, cytokine and chemokine profile of monocytes and macrophages at various states of macrophage polarization.

## Methods

### Cell culture

The human colorectal adenocarcinoma cell lines SW480 and SW620 used for EV production were purchased from the American Type Culture Collection (ATCC, USA). The cells were cultured in DMEM/F12 medium (Lonza, Germany) supplemented with 10% Fetal Bovine Serum (Sigma- Aldrich, F7524), 1% L-glutamine (Lonza, Germany) and 1% Antibiotic-Antimycotic (Gibco, USA) at 37 °C and 5% CO_2_ atmosphere.

The human monocytic leukemia cell line THP-1 was obtained from the European Collection of Authenticated Cell Cultures (ECACC 88081201). THP-1 cells were cultured in RPMI-1640 culture medium (Sigma Aldrich, Germany) supplemented with 10% FBS (Sigma-Aldrich, Germany) and penicillin/streptomycin 100 U/mL/100 μg/mL (Gibco, USA) as well as 25 mM HEPES (Sigma-Aldrich, Germany) (complete medium). Cells were propagated in 5% CO_2_ at 37 °C, 95% humidity.

The differentiation of THP-1 monocytes into inactive (M0) and then polarized (M1 and M2) macrophages was performed according to Genin et al., 2015 [19]. In brief, THP-1 monocytes were differentiated into M0 macrophages by incubation with 150 nM phorbol 12-myristate 13-acetate (PMA) (Calbiochem, USA) for 24 h followed by incubation in complete RPMI-1640 medium for another 24 h. M0 macrophages were then polarized into M1 macrophages by incubation with 10 pg/mL of bacterial lipopolysaccharides (LPS) (Sigma-Aldrich, Germany) and 20 ng/mL of interferon gamma (IFN-γ) (R&D Systems, USA) for 48 h. M2 polarization was induced by adding 20 ng/mL of interleukin-13 (IL-13) (R&D Systems, USA) and 20 ng/mL of interleukin-4 (IL-4) (R&D Systems, USA) to M0 macrophages for 48 h.

SW480 and SW620-derived EVs were added to THP1 monocytes (M) and inactive macrophages (M0) at 48 h time point at final concentration 10 μg/mL [see Additional file 1]. To analyze the effect of EVs on macrophage polarization into M1 and M2 subtypes SW480 and SW620-derived EVs were added to M0 macrophages prior to LPS + IFN-γ and IL-4 + IL-13 treatment. The morphology of activated macrophages was assessed by light microscopy using a transmitted light microscope EVOS XL (Advanced Microscopy Group, USA).

The cell cultures were monitored for mycoplasma infection using a PCR Mycoplasma Test Kit I/C (PromoKine, Germany).

### EV isolation

For EV isolation, SW480 and SW620 cells were seeded at 1 × 10^6^ cells/ mL in serum-free DMEM/F12 medium supplemented with 1% L-glutamine (Lonza, Germany), 10 ng bFGF (SantaCruz, Germany), 20 ng/mL EGF (R&D Systems, USA), 50 ng/mL hydrocortisone (Sigma-Aldrich, Germany), and 1xB27 (Invitrogen, USA), and cultured for 48 h. Culture medium was centrifuged at 300 × g to remove the cells and at 3000 × g to remove the cell debris and then filtered through 0.2 μm filters (Sarstedt, Germany). The medium was then concentrated up to 1 mL with 100 kDa centrifuge filters (Merck Millipore, Germany) and size exclusion chromatography (SEC) was performed to separate EVs from proteins by using CL6B sepharose (GE Healthcare, USA) filled columns (Kinesis, USA). Each SEC fraction was measured by Zetasizer Nano ZS and fractions containing from 30 to 200 nm particles in diameter were combined, concentrated to 100 μl in 3 kDa centrifuge filters (Merck, Millipore, Germany) and aliquoted to avoid repeated freeze/ thaw cycles. Isolated EVs were characterized by Western Blot (WB) and Transmission Electron Microscopy (TEM).

### Western blot

Cells and EVs were lysed in RIPA buffer (50 mM Tris, pH 8.0, 0.6 M NaCl, 4% Triton X-100, 2% sodium deoxycholate, 0.1% SDS). Ten micrograms of cellular and EV total protein were applied per lane and separated by 10% SDS-PAGE. Proteins were electroblotted onto nitrocellulose membranes and stained with Ponceau S solution to check the protein loading. The membranes were destained, blocked with 10% (*w*/*v*) fat-free milk and then incubated with the primary antibodies: CD9 (Santa Cruz, Germany) (1:500), ALIX (Santa Cruz, Germany) (1:1000), TSG101 (Abcam, UK) (1:1000), β-actin (Abcam, UK) (1:4000) and Calnexin (Abcam, UK) (1:1000). After washing, the membranes were incubated with peroxidase - conjugated rabbit anti-mouse secondary antibody (Santa Cruz, Germany) (1:2000) or mouse anti-rabbit secondary antibody (Santa Cruz, Germany) (1:2000), washed and processed with ECL Select Western Blotting Detection Reagents (GE Healthcare, USA) according to manufacturer’s instructions.

### EV labeling

The total protein concentration in EV preparations was measured using Pierce Coomassie (Bradford) Protein Assay Kit (Thermo Scientific, USA) according to the manufacturer’s instructions. EVs were labeled with Syto RNA Select (Invitrogen, USA) according to the manufacturer’s protocol. Briefly, 10 μg EVs were re-suspended in 100 μl of PBS per labeling reaction. One microliter of 1 mM Syto RNA Select dye stock solution was added to the EV sample obtaining a final dye concentration of 10 μM. The EVs and the dye were gently vortexed to obtain a homogenous distribution of the dye within the sample and incubated at 37 °C for 20 min. The unincorporated dye was then removed from the sample using Exosome Spin Columns MW 3000 (Invitrogen, USA) following the manufacturer’s protocol. For the dye control, labeling was performed as described but without EVs.

### EV uptake studies by flow cytometry

For the flow cytometry experiments, THP-1 monocytes (1 × 10^5^ cells per well in 100 μl of cell culture media) were seeded on 96-well cell culture plates (Sarstedt, Germany) and then incubated with Syto RNA Select-labeled SW480 and SW620-derived EVs for 1 h at 37 °C. First, the optimal EV concentration was determined by incubating THP-1 cells with Syto RNA Select-labeled EVs at concentrations 2 to 10 μg/mL, using 2 μg/mL increments. Then the EV uptake was quantitatively evaluated using 10 μg of EV total protein per mL. The EV uptake in THP-1 monocytes was performed in 5 biological replicates.

To study the EV uptake in M0 macrophages, 2.5 × 10^5^ THP-1 monocytes were seeded on 24-well cell culture plates (Sarstedt, Germany), treated with 150 nM PMA (Calbiochem, USA) for 24 h and incubated in RPMI-1640 complete medium for another 24 h as described above. Then M0 macrophages were incubated with Syto RNA Select-labeled SW480 and SW620 EVs at concentration 10 μg/mL for 1 h at 37 °C. The EV uptake in M0 macrophages was performed in 4 biological replicates.

Following incubation, cells were collected for flow cytometry analysis by aspirating from plate (THP-1 monocytes) or by detaching with Accutase (M0 macrophages, Gibco, USA) and, after washing the cell samples with PBS, flow cytometry was performed on a Guava EasyCyte cytometer (Merck Millipore, Germany) with 10,000 events recorded per sample to measure Syto RNA Select fluorescence intensity. Flow cytometry data was analyzed using FlowJo software, version 10 (Tree Star Inc., USA).

### EV uptake in THP-1 monocytes by fluorescence microscopy

THP-1 monocytes (1 × 10^5^ cells per well in 100 μl of cell culture media) were seeded on 96-well cell culture plates (Sarstedt) and incubated with 10 μg/mL EVs for 1 h at 37 °C. Following incubation, the cells were washed in 1 mL PBS and centrifuged at 2000 rpm for 5 min. The cells were then fixed with 4% paraformaldehyde solution at room temperature for 20 min and washed twice with 1 mL PBS. Fixed cells were stained for F-actin filaments with ActinRed 555 ReadyProbes reagent (Invitrogen, USA), and cell nuclei were counterstained with Hoechst 33,342 (Invitrogen, USA). Following staining, samples were washed three times with 1 mL PBS. Stained cells were smeared on adhesion microscope slides (Marienfeld, Germany), air-dried at room temperature for 10 min to remove excess water and then mounted with ProLong Diamond (Invitrogen, USA). Mounted cells were visualized under a Nikon C2 microscope using a FITC filter for Syto RNA Select, a TRITC filter for ActinRed 555 ReadyProbes reagent and a DAPI filter (Nikon, Japan) for Hoechst 33,342. Each channel was recorded separately to avoid spectral overlap. The images were analyzed using Nis-Elements C 4.13 software (Nikon, Japan). The EV uptake by fluorescence microscopy was studied in 3 biological replicates.

### EV uptake pathway analysis

For the EV uptake pathway analysis, 30 min prior to adding Syto RNA Select-labeled SW480 and SW620 EVs, THP-1 monocytes and M0 macrophages were pretreated with selected uptake inhibitors. Then, Syto RNA Select-labeled SW480 and SW620 EVs were added to the cell cultures at the final concentration 10 μg/mL and allowed to incubate for 1 h at 37 °C. The following inhibitors targeting clathrin-dependent endocytosis (80 μM dynasore hydrate, 10 μM chlorpromazine), caveolae and/or lipid raft-dependent endocytosis (20 μM nystatin) and macropinocytosis/phagocytosis (20 μM cytochalasin D and 5 μM 5-ethyl-N-isopropyl amiloride (EIPA)) were used [2, 20–24]. However, it should be mentioned that even if some these inhibitors are claimed to affect individual endocytic pathways, it is possible that they may affect other endocytic pathways to some extent [25]. With the exception of EIPA that was from Cayman Chemical, USA, the other inhibitors were from Sigma-Aldrich, Germany. Non-toxic inhibitor concentrations were selected according to results obtained in the cell counting kit 8 (CCK-8) assay (Sigma Aldrich, Germany).

Following incubation, cells were either aspirated (THP-1 monocytes) or detached (M0 macrophages) from plates, washed with 1 mL PBS (Amresco, USA), centrifuged at 2000 rpm for 5 min (Mikro 120, Hettich Zentrifugen, Germany) and then re-suspended in 200 μl of PBS. Flow cytometry was performed on a Guava EasyCyte cytometer (Merck Millipore, Germany) with 10,000 events recorded per each sample to measure Syto RNA Select fluorescence intensity. Flow cytometry data was analyzed using FlowJo software, version 10 (Tree Star Inc., USA). EV uptake pathway analysis was performed in 4 biological replicates (THP-1 monocytes) or in 2 biological replicates (M0 macrophages).

### Cell viability assay

The impact of SW480 and SW620 EVs on the viability of THP-1 monocytes and macrophages was analyzed using the Cell Counting Kit 8 (CCK-8) (Sigma-Aldrich, USA**)**. A total of 5 × 10^4^ THP-1 monocytes per well were seeded onto 96-well plates (Sarstedt, Germany) in 100 μL of complete RPMI 1640 medium and exposed to macrophage differentiation protocol as previously described. After two days, EVs at final concentration 10 μg/mL were added, and the cells were incubated with EVs for the next 48 h. EV-untreated cells of each macrophage subset were used as a control, and the viability of control cells was defined as 100%. Following incubation, 10 μL of CCK-8 reagent was added to each well and incubated for 3 h at 37 °C in 5% CO_2_ at 95% humidity. The optical density was measured using a spectrophotometer Bio-Tek *ELx808* (BioTek Instruments, USA) at a wavelength of 450 nm. The background signal was subtracted from all the samples.

Cell viability was calculated using the following formula:

$$ Viability,\%=100\ \mathrm{x}\ \frac{\mathrm{OD}450\ \mathrm{of}\ \mathrm{the}\ \mathrm{cells}\ \mathrm{treated}\ \mathrm{with}\ \mathrm{EVs}}{\mathrm{OD}450\ \mathrm{of}\ \mathrm{untreated}\ \mathrm{control}\ \mathrm{cells}} $$

where OD450 is the optical density at 450 nm. EV cytotoxicity was calculated by subtracting the calculated viability of EV-treated cells from the viability of untreated control cells of the respective macrophage subset. Data were analyzed using Microsoft Excel and GraphPad Prism software.

### Cell immunophenotyping by flow cytometry

Cells were either aspirated (monocytes as suspension cells) or detached from the plate surface with StemPro Accutase (Gibco, USA). Then samples were washed with 1 mL PBS and centrifuged at 1200 rpm (135 x g) for 5 min (Mikro 120, Hettich Zentrifugen, Germany) and re-suspended in 100 μl of 1% FBS in PBS. For CD68 detection, cells were fixed with Cytofix, then permeabilized and washed with PermWash (BD Biosciences, Switzerland). Fc receptor block (BD Pharmingen, Switzerland) was applied for 10 min at room temperature prior to antibody staining. The following antibodies with the corresponding isotype controls were selected for flow cytometry analysis: CD14 PE, CD206 PE, HLA-DR PerCP-Cy5.5 and CD68 FITC (BD Pharmingen, Switzerland). Antibody conjugates were incubated with samples containing 1 × 10^5^ cells in 100 μL of staining buffer containing 1% FBS in PBS in microcentrifuge tubes for 30 min at room temperature. Flow cytometry measurements were performed on a Guava EasyCyte cytometer (Merck Millipore, Germany) with 10,000 events recorded per each sample. Flow cytometry data was analyzed using FlowJo software, version 10 (Tree Star Inc., USA). Cell immunophenotyping of EV treated monocytes and macrophages was performed in 4 biological replicates. Cell immunophenotyping of EV-untreated cells was performed in 7 biological replicates.

The effect of the endocytosis inhibitor dynasore hydrate on CD14 expression in M0 macrophages was analyzed by pre-treating M0 macrophages with 80 μM dynasore hydrate for 30 min and then incubating with EVs at final concentration 10 μg/mL for 1 h at 37 °C. M0 macrophages were then cultivated in complete RPMI-1640 cell culture medium for another 48 h and CD14 expression was analyzed by flow cytometry.

### Studies of cytokine secretion patterns in monocytes and polarized macrophages

Cell culture supernatants were aspirated from plates with THP-1 monocytes differentiated in the presence or absence of EVs, centrifuged and stored at − 80 °C until further analysis. The cytokine content was assessed using a Magnetic Luminex Assay kit (R&D Systems, USA). A panel of eight cytokines and chemokines (CXCL10, MMP-9, IL-1 β, TNFα, IL-6, IL-23, IL-10 and CCL22) was selected for analysis and measured in a Luminex 200 analyzer instrument (Merck Millipore, Germany) according to the manufacturer’s protocol. Briefly, cell culture supernatants were diluted 2-fold and incubated with antibody-coated microparticles for 2 h at room temperature. After a washing step, samples were incubated with biotinylated antibodies. Following a wash, streptavidin-phycoerythrin conjugate was added. After the final wash, the microparticles were resuspended in the assay buffer and analyzed in a Luminex 200 detection platform. Luminex data were analyzed using Prism software (GraphPad Software Inc., USA). To establish the method and to ensure the reproducibility of the Luminex analysis, cytokine secretion patterns of EV-untreated cells were studied in 5 biological replicates in total. Cytokine secretion patterns of EV-treated cells were studied in 3 biological replicates.

The effect of dynasore hydrate on *CXCL10* and *IL-10* gene expression in M0 macrophages was analyzed by pre-treating M0 macrophages with 80 μM dynasore hydrate for 30 min and then incubating with EVs at final concentration 10 μg/mL for 1 h at 37 °C. M0 macrophages were then cultivated in complete RPMI-1640 cell culture medium for another 48 h and gene expression was analyzed by quantitative PCR method. Cells were lysed using QIAzol lysis reagent. Total cellular RNA was extracted from cells according to the manufacturer’s guidelines (Qiagen, Germany). The concentration and purity of RNA were determined using a Tecan Infinite M200 Pro microplate reader (Switzerland). The RNA concentration was normalized to 1 μg/μl for all samples.

Extracted RNA was used for cDNA synthesis by FIREScript RT cDNA Synthesis Kit according to the manufacturer’s instructions. Real-time RT-PCR was performed for quantitative gene expression analysis using 5× HOT FIREPol EvaGreen qPCR Mix Plus (ROX) according to the manufacturer’s instructions. All reagents were obtained from Solis BioDyne (Estonia). Ct values were normalized to the average Ct value of the housekeeping gene *GAPDH*. Fold change in gene expression was calculated using the ΔΔCt method. Data were expressed as –ΔΔCt relative fold change. Primer sequences: CXCL10-F 5’-GAACCTCCAGTCTCAGCACC-3′, CXCL10-R 5’-GAGAGGTACTCCTTGAATGCCA-3′, IL10-F 5’CCTGCCTAACATGCTTCGAG-3′, IL10-R 5’CAACCCAGGTAACCCTTAAAGTC-3′.

### Statistical analysis

Statistical analysis was performed using GraphPad Prism Software (GraphPad Inc., California, USA). Data were expressed as mean ± standard deviation (SD) or mean with range. Differences between studied groups (*n* ≥ 2) were statistically assessed by t-test and ANOVA as indicated. Significance was defined at *p ≤ 0.05, **p ≤ 0.01, ***p ≤ 0.001 and **** ≤ 0.0001.

## Results

### EV isolation and characterization

**Fig. 1:**
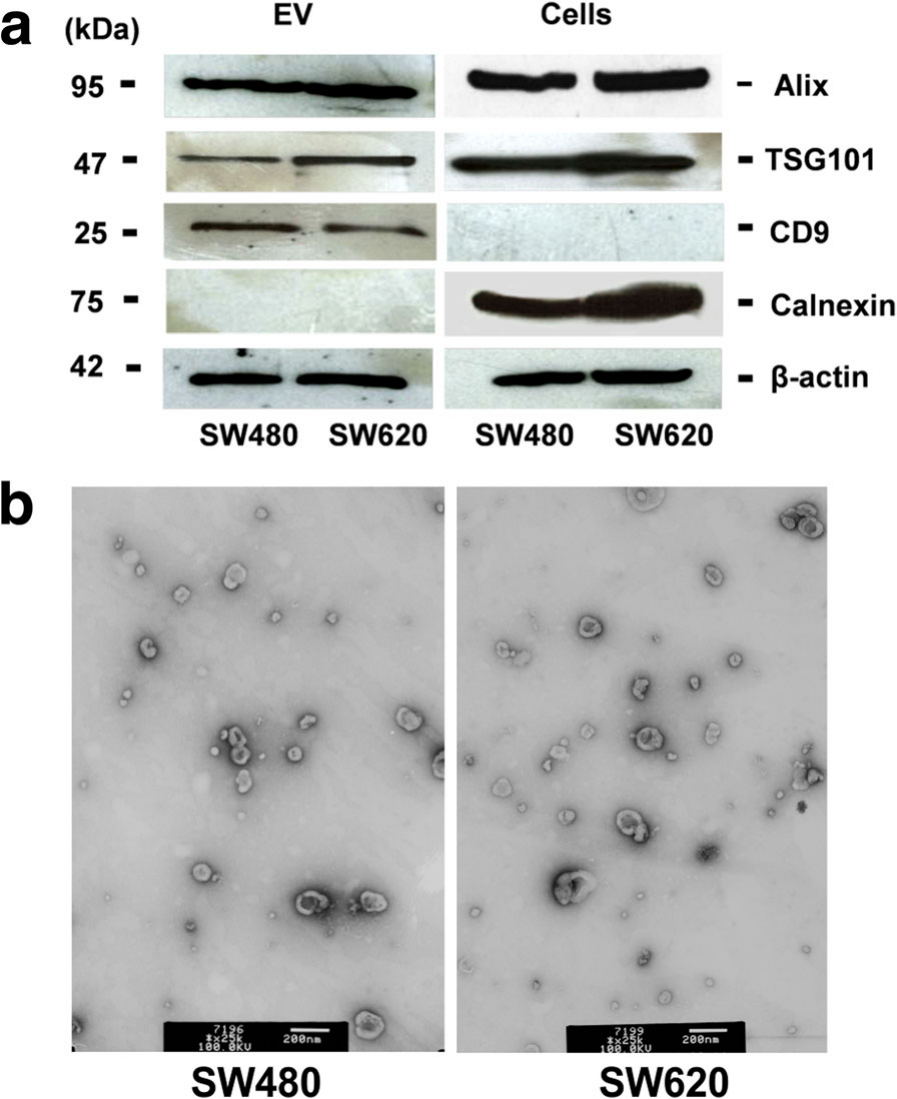
Characterization of SW480 and SW620-derived EVs. **a** Western blot analysis of EVs markers (ALIX, TSG101 and CD9), loading control (β-actin) and negative control (Calnexin) in EVs. **b** Transmission electron microscopy image of EVs showing the size range. White scale bar represents 200 nm

EVs were isolated by sequential centrifugation, filtration and SEC from the conditioned medium of the human isogenic CRC cell lines SW480 and SW620 cultured for 48 h. The isolated EVs were characterized by Western blot analysis and, as shown in Fig. 1a, EVs from both SW480 and SW620 cells contained the EV-associated markers ALIX (PDCD6IP), TSG101 and CD9. EVs were negative for the endoplasmic reticulum protein Calnexin, thus confirming that the EV preparations were not contaminated to a large extent with cellular components (Fig. 1a). TEM revealed that the particles have the cup-shaped morphology typically observed with the protocol we used and were ranging in size from 30 to 130 nm (Fig. 1b).

### THP-1 monocytes and M0 macrophages take up SW480 and SW620-derived EVs

**Fig. 2:**
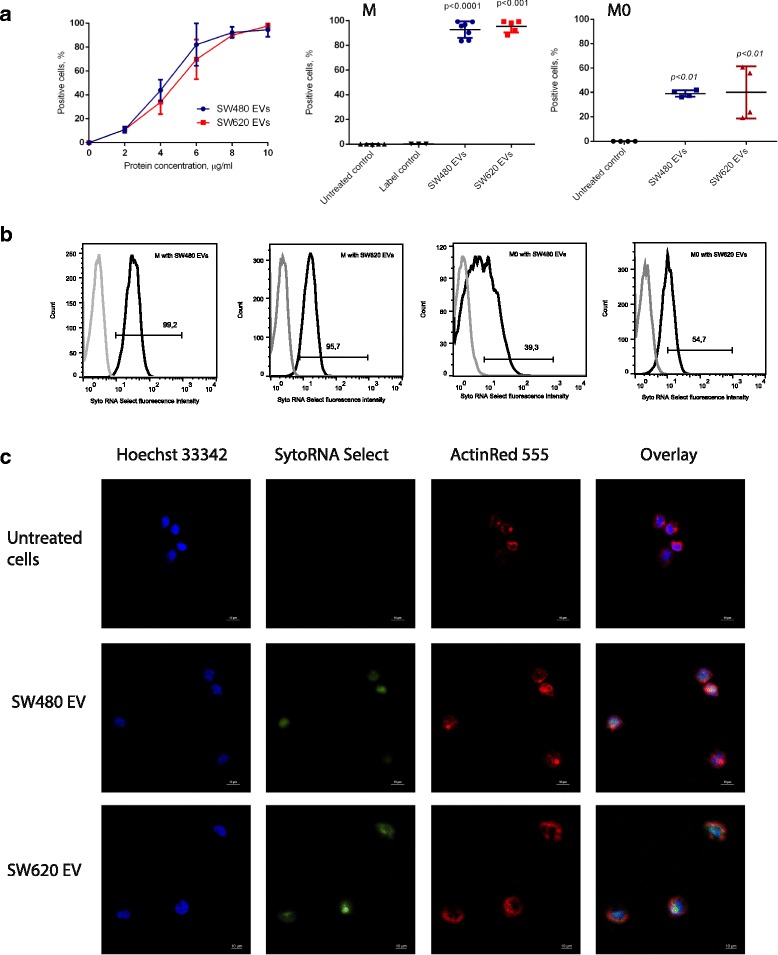
SW480 and SW620-derived EV uptake in THP-1 monocytes and M0 macrophages. **a** Flow cytometry analysis showing concentration-dependent uptake of Syto RNA select labelled EVs by THP-1 monocytes. The graphs show the percentage of Syto RNA select-positive THP-1 monocytes in relation to EV concentration (left) and Syto RNA Select-positive THP-1 monocytes (M, middle) and M0 macrophages (M0, right) following incubation with Syto RNA Select labelled EV at final concentration 10 μg/mL. Data are shown as mean ± SD (*n* = 5). Statistical analysis was carried out with one-way ANOVA test. **b** Representative flow cytometry histograms showing Syto RNA Select labelled SW480 and SW620 EV uptake in THP-1 monocytes and M0 macrophages (*n* ≥ 4). Grey lines represent untreated cells; black lines represent SW480 or SW620 EV (10 μg/mL) treated monocytes (M) or macrophages (M0). Histogram bar shows the percentage of Syto RNA select positive cells in the respective analysis. **c** Representative fluorescence microscopy images showing Syto RNA select labelled SW480 EV and SW620 EV uptake in THP-1 monocytes (n = 3). THP-1 monocytes were incubated with Syto RNA select labelled SW480 or SW620 EVs (10 μg/mL) for 1 h (green). The cytoskeleton was labelled with F-actin probe ActinRed 555 (red). The nuclei were stained with Hoechst 33,342 (blue). Scale bar is 10 μm

First, the optimal amount of EVs for uptake experiments was determined by incubating THP-1 cells with Syto RNA Select labeled EVs at concentrations from 2 to 10 μg/mL for 1 h at 37 °C. As shown in Fig. 2a, the percentage of Syto RNA Select positive cells increased when increasing protein concentrations of EVs were added. For SW480 EVs, a plateau was reached at 8 μg/mL with more than 90% cells positive for Syto RNA Select label. Similarly, approximately 85% of SW620 EV treated cells were Syto RNA Select positive after incubation with 8 μg/mL EVs, and the maximum uptake was reached at 10 μg/mL. Based on these results, an EV concentration of 10 μg/mL was selected for further experiments. The uptake efficiency of SW480 and SW620 EVs at 10 μg/mL in THP-1 monocytes (M) was confirmed by flow cytometry analysis (Fig. 2a, b). Regarding cell survival following EV treatment, we observed induction of THP-1 monocyte proliferation by SW480 and SW620 EVs. M0 macrophage proliferation was induced by SW480 EVs [see Additional file 2 a]. There was a slight cytotoxicity observed in M0 and M1 macrophages after incubation with SW620 EVs [see Additional file 2 b]. Nevertheless, the cell viability was within acceptable range for the accomplishment of the experiments.

Additionally, the uptake and intracellular localization of SW480 and SW620 derived EVs in THP-1 monocytes was studied by fluorescence microscopy. After 1 h incubation, the Syto RNA Select signal could be detected in the cell cytoplasm indicating that SW480 and SW620 cell-derived EVs had been internalized in THP-1 monocytes (Fig. 2c). We also estimated the EV uptake efficiency in M0 macrophages differentiated according to the THP-1 monocyte differentiation procedure [see Additional file 1]. Syto RNA Select-labeled SW480 and SW620 EVs (10 μg/mL) were incubated with M0 macrophages for 1 h at 37 °C and analyzed for uptake efficiency by flow cytometry. As shown in Fig. 2a, b, the EV uptake efficiency in M0 macrophages was on average 40% of cells after 1 h incubation for both SW480 and SW620 EVs.

### SW480 and SW620-derived EVs enter THP-1 monocytes and M0 macrophages via a dynamin-dependent endocytic pathway

To study the uptake mechanism of SW480 and SW620 EVs by THP-1 monocytes and M0 macrophages, the cells were incubated with Syto RNA Select labelled EVs in the presence of endocytosis inhibitors and then assessed for Syto RNA Select fluorescence intensity by flow cytometry. Based on the literature analysis, the following inhibitors affecting different endocytosis pathways were selected: dynasore hydrate, chlorpromazine (targeting clathrin-dependent endocytosis), nystatin (caveolae and/or lipid raft-dependent endocytosis inhibitor), cytochalasin D and EIPA (micropinocytosis and phagocytosis inhibitors) [2, 20–24].The optimal inhibitor concentration was tested by cell counting 8 (CCK-8) assay and the non-cytotoxic concentrations of 80 μM dynasore hydrate, 10 μM chlorpromazine, 20 μM cytochalasin D, 20 μM nystatin and 5 μM 5-ethyl-N-isopropyl amiloride (EIPA) were selected for uptake pathway analysis (data not shown).

**Fig. 3:**
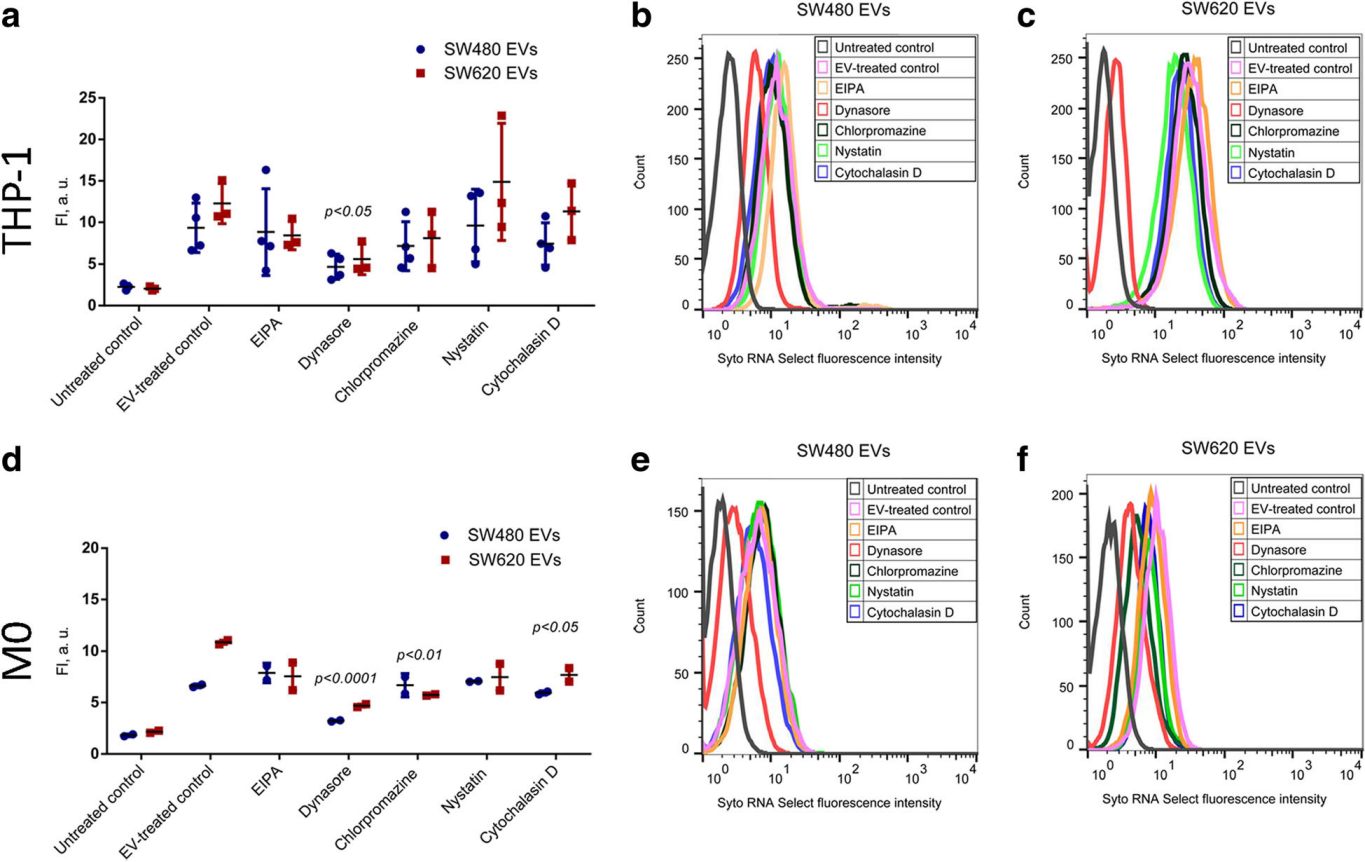
SW480 and SW620-derived EV uptake pathway studies in THP-1 monocytes and M0 macrophages by flow cytometry analysis. **a** Fluorescence intensity of THP-1 monocytes following incubation with Syto RNA select labelled EV (10 μg/mL) in the presence of uptake inhibitors: 5 μM 5-ethyl-N-isopropyl amiloride (EIPA), 80 μM dynasore hydrate, 10 μM chlorpromazine, 20 μM nystatin and 20 μM cytochalasin D. Untreated cells were used as negative control and EV-treated cells served as positive control. The graph represents mean ± SD (n = 3). **b, c** Flow cytometry histograms showing fluorescence intensity of THP-1 monocytes following incubation with 10 μg/mL of Syto RNA Select labeled SW480 EVs (b) or SW620 EVs (c) in the presence or absence of the uptake inhibitors. Images are representative of 3 biological replicates. **d** Flow cytometry analysis of M0 macrophages following incubation with Syto RNA select labelled SW480 and SW620 EVs (10 μg/mL) in the presence of uptake inhibitors: 5 μM 5-ethyl-N-isopropyl amiloride (EIPA), 80 μM dynasore hydrate, 10 μM chlorpromazine, 20 μM nystatin and 20 μM cytochalasin D. Untreated M0 cells were used as a negative control and EV-treated M0 cells served as positive control. The graph represents mean ± SD (*n* = 2). **e, f** Flow cytometry histograms showing fluorescence intensity of M0 macrophages following incubation with 10 μg/mL of Syto RNA Select labeled SW480 EVs (e) or SW620 EVs (f) in the presence or absence of uptake inhibitors. Statistical analysis carried out with a two-way ANOVA test followed by Sidak’s post-test. *p ≤ 0.05 vs. EV-treated cells of the respective monocyte-macrophage cell subset

In THP-1 monocyte cells incubated with Syto RNA Select-labeled SW480 EVs and SW620 EVs, the fluorescence intensity (FI) was reduced by 49.8% and 54.6%, respectively, by dynasore hydrate (Fig. 3a-c). In M0 macrophages, the uptake of SW480 and SW620 EVs was also inhibited by dynasore hydrate (FI was reduced by 53.3% and 56% respectively) (Fig. 3d-f). Additionally, the uptake of SW620 cell-derived EVs in M0 macrophages was inhibited by chlorpromazine (45.6%) and cytochalasin D (27.9%) (Fig. 3d-f). Other inhibitors slightly reduced EV uptake but without reaching significance (Fig. 3a, d). Control experiments showed that the EV uptake in THP-1 monocytes was abrogated completely at + 4 °C [see Additional file 3].

### CRC EVs alter monocyte-macrophage surface marker expression

**Fig. 4:**
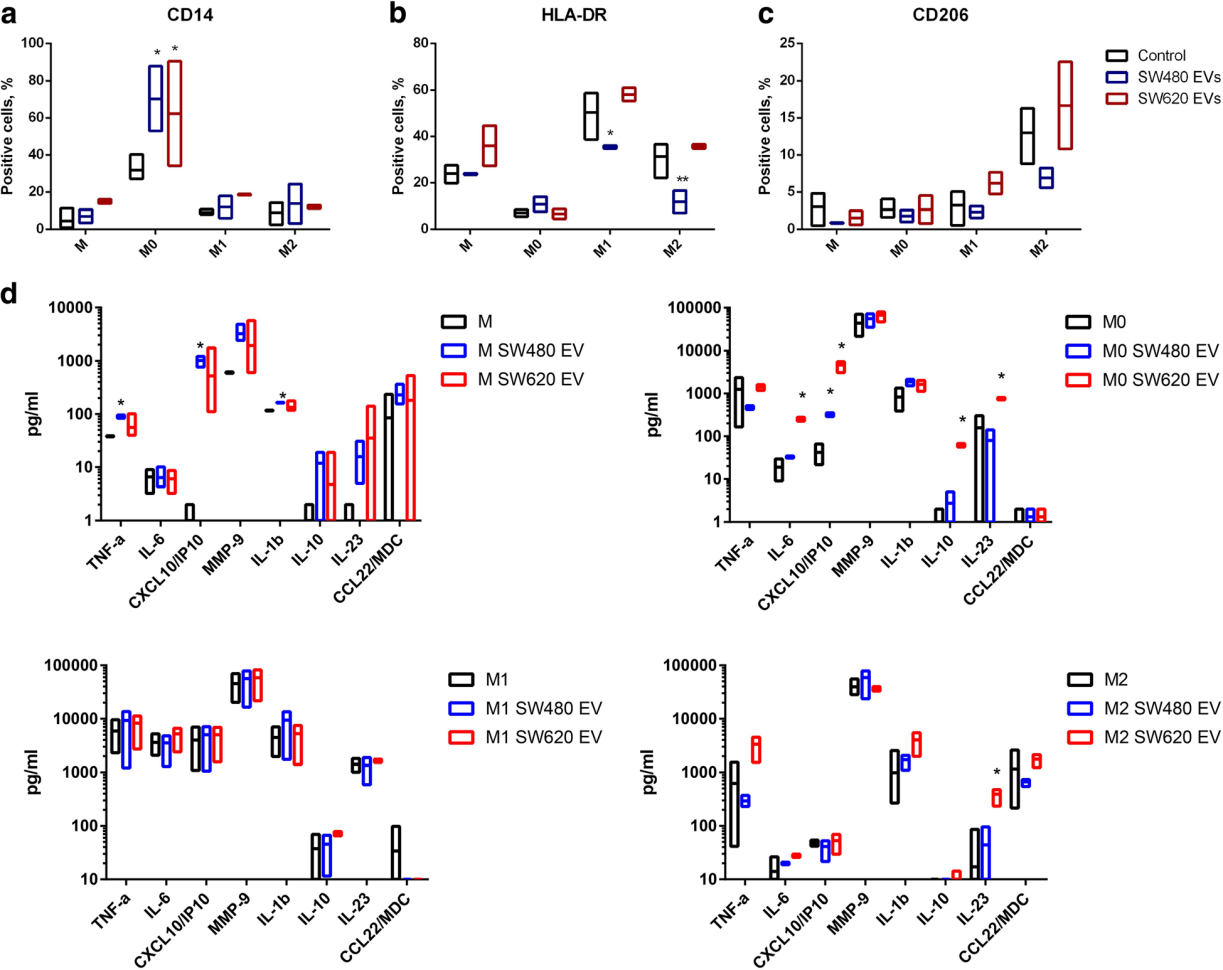
SW480 and SW620-derived EV effect on immunophenotype and cytokine secretion of THP-1 monocytes (M), inactive macrophages (M0) and polarized macrophages (M1 and M2). **a, b, c** Flow cytometry analysis showing the percentage of CD14, HLA-DR and CD206 positive cells (mean with range, n = 4) at M, M0, M1 and M2 stages following incubation with 10 μg/mL of SW480 EVs or SW620 EVs. Statistical analysis was carried out with two-way ANOVA test. *p ≤ 0.05; **p ≤ 0.01 vs. untreated controls of the respective monocyte-macrophage cell subset. **d** Cytokine and chemokine secretion pattern in M, M0, M1 and M2 stages following incubation with 10 μg/mL of SW480 EVs or SW620 EVs analysed by Luminex assay (mean with range, n = 3). Statistical analysis was carried out with multiple t-tests using Holm-Sidak method for multiple comparison correction. *p ≤ 0.05 vs. untreated controls of the respective monocyte-macrophage cell subtype

In this study, the model established by Genin et al., 2015 for monocyte to macrophage differentiation and polarization was used [see Additional files 1, 4]. The cell surface markers CD14, HLA-DR and CD206 were chosen for analysis: CD14 is a pattern recognition receptor for several ligands, primarily for bacterial LPS [26], HLA-DR is necessary for antigen presentation [27, 28], whereas CD206 is a mannose receptor that is considered a typical M2 macrophage marker [19]. Exposure to SW480 and SW620 EVs notably increased CD14 expression in M0 macrophages (Fig. 4a). Furthermore, SW480 EVs decreased HLA-DR expression in M1 and M2 macrophages (Fig. 4b). Exposure to EVs did not cause statistically significant changes in CD206 expression in any of the macrophage subsets (Fig. 4c).

**Fig. 5:**
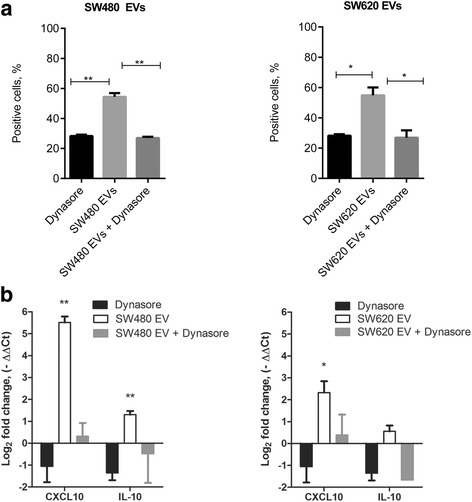
Effect of dynasore hydrate on the SW480 and SW620 EV-induced changes on the expression of the surface marker CD14 and on the gene expression of *CXCL10* and *IL-10* in M0 macrophages. **a** Flow cytometry analysis showing the percentage of CD14-positive M0 macrophages. The graphs represent mean ± SD (n = 2). Statistical analysis was carried out with t-test. *p ≤ 0.05, **p ≤ 0.01 **b** qPCR analysis showing changes in *CXCL10* and *IL-10* gene expression (n = 3).

In addition, we observed that dynasore hydrate blocked the EV effect on CD14 expression in M0 cells (Fig. 5a).

### EV effect on the cytokine and chemokine secretion profile of monocytes and macrophages

A panel of eight pro- and anti-inflammatory cytokines and chemokines representing the M1 type of response (CXCL10, IL-23, IL-1 β, TNFα, IL-6 and MMP-9) and the M2 type of response (CCL22, IL-10) was selected to evaluate the effect of CRC cell line derived EVs on the monocyte and macrophage secretory profiles. We observed pronounced cytokine and chemokine secretion alterations in THP-1 monocytes and M0 macrophages following exposure to SW480 and SW620 EVs. Exposure to SW480 cell-derived EVs significantly increased CXCL10, TNFα and IL-1β levels in monocytes, whereas in inactive M0 macrophages CXCL10 production was induced (Fig. 4d) [see Additional file 5]. We observed that exposure to SW480 EVs had no significant effect on cytokine production in M1 or M2 polarized macrophages (Fig. 4d). Furthermore, SW620 EVs induce mixed M1 (CXCL10, IL-6, IL-23) and M2 (IL-10) cytokine secretion pattern in inactive M0 macrophages and IL-23 production in M2 polarized macrophages (Fig. 4d, M0 and M2 plots). Additionally, we show that dynasore hydrate inhibits the effect of EVs on the expression of *CXCL10* and *IL-10* mRNA confirming that cytokine and chemokine induction in M0 macrophages results from EV treatment (Fig. 5b).

## Discussion

In the current study, we have analyzed the internalization and functional effects of two isogenic CRC cell line SW480 and SW620 derived EVs on monocytes and macrophages using the human THP-1 monocyte differentiation model [19, 29]. Our data show that THP-1 monocyte cells internalize SW480 and SW620 derived EVs and that EV uptake was inhibited by dynasore. This compound is an inhibitor of dynamin, although it may have dynamin-independent effects [30]. Dynamin has been involved in several types of endocytosis such as clathrin-mediated endocytosis, macropinocytosis and caveolae-mediated endocytosis. Moreover, in M0 macrophages the SW480 and SW620 EV uptake was inhibited by dynasore and chlorpromazine (an inhibitor of clathrin-mediated endocytosis). Additionally, SW620 EV uptake in M0 macrophages was inhibited by cytochalasin D, which is an inhibitor or macropinocytosis and phagocytosis. These results show that the endocytosis of SW480 and SW620 derived EVs in THP-1 monocytes and M0 macrophages occurs via a dynamin-dependent endocytic pathways and phagocytosis. However, further experiments are required to determine the specific endocytic pathway(s) that are involved in CRC cell-derived EV uptake. Interestingly, Feng *et al.* have previously shown that cancer cell-derived EVs are internalized in monocytes via phagocytosis [20].

Next, we analyzed the effect of EVs derived from these two CRC cell lines on an established monocyte-to-macrophage differentiation model. First, the monocyte to macrophage differentiation model was established as described by Genin et al. [19]. THP-1 monocytes were differentiated by PMA into M0 macrophages, which are characterized by an adherent phenotype and expression of CD14 [see Additional file 1]. Next, M0 macrophages were polarized into M1 macrophages in the presence of LPS and IFN-γ. M1 macrophages are characterized by an adherent phenotype and HLA-DR expression [see Additional file 1]. Finally, the polarization into M2 macrophages was carried out in the presence of IL-4 and IL-13. The M2 phenotype was confirmed by a fibroblast-like extended morphology and CD206 expression [see Additional file 1]. M1 macrophages secreted IL-6, CXCL10, IL-10 and IL-23, whereas M2 macrophages produced CCL22. Both M1 and M2 polarized macrophages secreted IL-1β, TNFα and MMP9 [see Additional file 4].

Using this model, our experiments show that exposure to CRC cell line derived EVs increased the expression of the surface marker CD14 in M0 macrophages. Noteworthy, it has been reported that an increase in the frequency of CD14^+^CD169^+^ cells may be associated with the development and progression of CRC and a concomitant rise of both pro-tumor (M2) and anti-tumor (M1) monocytes and infiltrating macrophages [31]. Primary CRC SW480 cell line-derived EVs increased CXCL10, TNF-α and IL-1β secretion in monocytes and CXCL10 secretion in M0 macrophages. Interestingly, elevated serum levels of CXCL10 have been associated with liver metastasis and poor survival in CRC [32]. There are several studies suggesting that increased CXCL10 secretion in the TME is associated with cancer cell invasive properties and metastasis formation [33]. The mechanisms suggested are increased expression of matrix-degrading enzymes, migration and adhesion that is attributable to CXCL10 [33]. In monocytes, SW480-derived EVs stimulated TNFα expression. This result is in line with findings by Stanilov et al. 2011, who observed that monocytes from patients with advanced cancer secreted significantly more TNFα than monocytes from patients at an early stage of the disease [34]. Moreover, an increased pretreatment level of the pro-inflammatory cytokines IL-1β, IL-6 and TNFα correlated with CRC progression [35].

Our results show that SW620 cell line-derived EVs induce a mixed M1 or pro-inflammatory (CXCL10, IL-6, and IL-23) and M2 or anti-inflammatory (IL-10) cytokine secretion pattern in inactive (M0) macrophages. Increased CXCL10 and IL-6 levels in patient blood have been associated with advanced CRC stage and IL-6 has been suggested as an independent adverse prognostic marker of survival [32, 36]. Additionally, an increased pre-operative serum IL-10 level correlates with poor survival in CRC patients [37, 38]. It has been reported that during intestinal inflammation macrophage produced IL-23 induce the production of the pro-inflammatory cytokines IL-6 and IL-17. Moreover, Treg and Th17 cells are among the IL-23 target cells. IL-23 is essential for the differentiation of Th17 lymphocytes, and the induction of IL-23 in CRC has been linked to a more aggressive disease [36]. Therefore, it is possible that CRC EVs might have implications for cancer progression through Th17 cell activation [39].

Our results suggest that exposure of monocytes and M0 macrophages to CRC EVs may contribute to monocyte migration towards the TME and induction of a pro-inflammatory response by TAM. Both CRC EV types induce significant alterations in the monocyte and inactive macrophage secretory profile. Additionally, we have observed an increased CD14 expression in M0 macrophages by both SW480 and SW620 EVs and a decreased HLA-DR expression in M1 and M2 polarized macrophages by SW480 derived-EVs in our model.

In this study we have analysed the effect of CRC derived EVs on monocytes and macrophages using a well-established THP-1 differentiation model. However, further validation of data using primary monocytes and *in vitro* generated macrophages derived from peripheral blood would be necessary to allow a general conclusion about the effects of colorectal cancer-derived EVs on the phenotype and secretory profile of macrophages. Additionally, more colon cancer cell lines and normal colon epithelium cell lines could be included in the study to conclude on the impact of CRC-derived EV on monocytes and macrophages.

## Conclusion

Based on these experiments we conclude that primary CRC-derived EVs modulate the immunophenotype and secretory profile of monocytes and inactive macrophages towards M1 type of response whereas metastatic CRC-derived EVs induce a mixed M1 and M2 cytokine response in inactive macrophages in the THP-1 monocyte differentiation model. Furthermore, although CRC EVs decrease HLA-DR expression in M1 and M2 polarized macrophages, their effect on the secretory profile of these cells is negligible.

## Additional files

Additional file 1: Experimental design of the THP-1 monocyte to macrophage differentiation showing the time points for the addition of EVs and stimulatory molecules. Below the experimental design, representative light microscopy images show morphology of THP-1 monocytes (M), M0 macrophages (M0), M1 macrophages (M1) and M2 macrophages (M2) (*n* = 4). Scale bar 100 μm. Representative flow cytometry dot plots show CD14, HLA-DR, CD206 and CD68 marker expression at M, M0, M1 and M2 stages. (JPG 1845 kb)

Additional file 2: SW480 and SW620-derived EV effect on monocyte (M) and macrophage (M0, M1, M2) viability. **a** OD values at 450 nm which are in direct proportion of viable cell counts. **b** SW480 and SW620 EV cytotoxicity on THP-1 monocytes and M0, M1 and M2 macrophages. The graphs represent mean ± SEM (*n* = 3). Statistical analysis carried out with the t-test. *p ≤ 0.05, **p ≤ 0.01 vs. untreated cell control of the respective monocyte-macrophage cell subset. (PDF 50 kb)

Additional file 3: Effect of temperature on the SW480 EV uptake in THP-1 monocytes. Flow cytometry histograms showing Syto RNA Select fluorescence intensities of untreated (left) and Syto RNA Select-labeled SW480 EV-treated THP-1 monocytes following incubation at 4 °C (middle) and 37 °C (right). Histogram markers show the percentage of Syto RNA Select-positive cells. (PDF 53 kb)

Additional file 4: TNFα, IL-23, IL-6, IL-1 β, CXCL10, CCL22, IL-10 and MMP9 secretion profile at different monocyte-macrophage differentiation stages. The graphs represent average biomolecule concentrations SEM (*n* = 3). Statistical analysis carried out with one-way ANOVA test. *p ≤ 0.05, **p ≤ 0.01, ***p ≤ 0.001 and **** ≤ 0.0001 vs. untreated cell control of the respective monocyte-macrophage cell subset. (PDF 63 kb)

Additional file 5: Effect of SW480 and SW620-derived EVs on biomolecule secretion patterns of monocytes and M0, M1 and M2 macrophages. Luminex data analysis showing TNFα, IL-6, CXCL10, IL-23, IL-10, MMP9, IL-1β and CCL22 concentration in cell culture supernatants of monocytes (M) and M0, M1 and M2 macrophages following incubation with SW480 and SW620 EVs or without them (control). The graphs represent mean ± SD (*n* = 3). (PDF 39 kb)

## Abbreviations.

CRC: Colorectal cancer
EIPA: 5-ethyl-N-isopropyl amiloride
EV: Extracellular vesicle
FI: Fluorescence intensity
PMA: Phorbol-12-myristate-13-acetate
SEC: Size-exclusion chromatography
TAM: Tumor-associated macrophage
TME: Tumor microenvironment
