# Supplementary figures and images for: Effect of colorectal cancer-derived extracellular vesicles on the immunophenotype and cytokine secretion profile of monocytes and macrophages
Source: Cell Commun Signal. 2018 Apr 24;16:17. doi: 10.1186/s12964-018-0229-y (PMC5937830; doi:10.1186/s12964-018-0229-y)

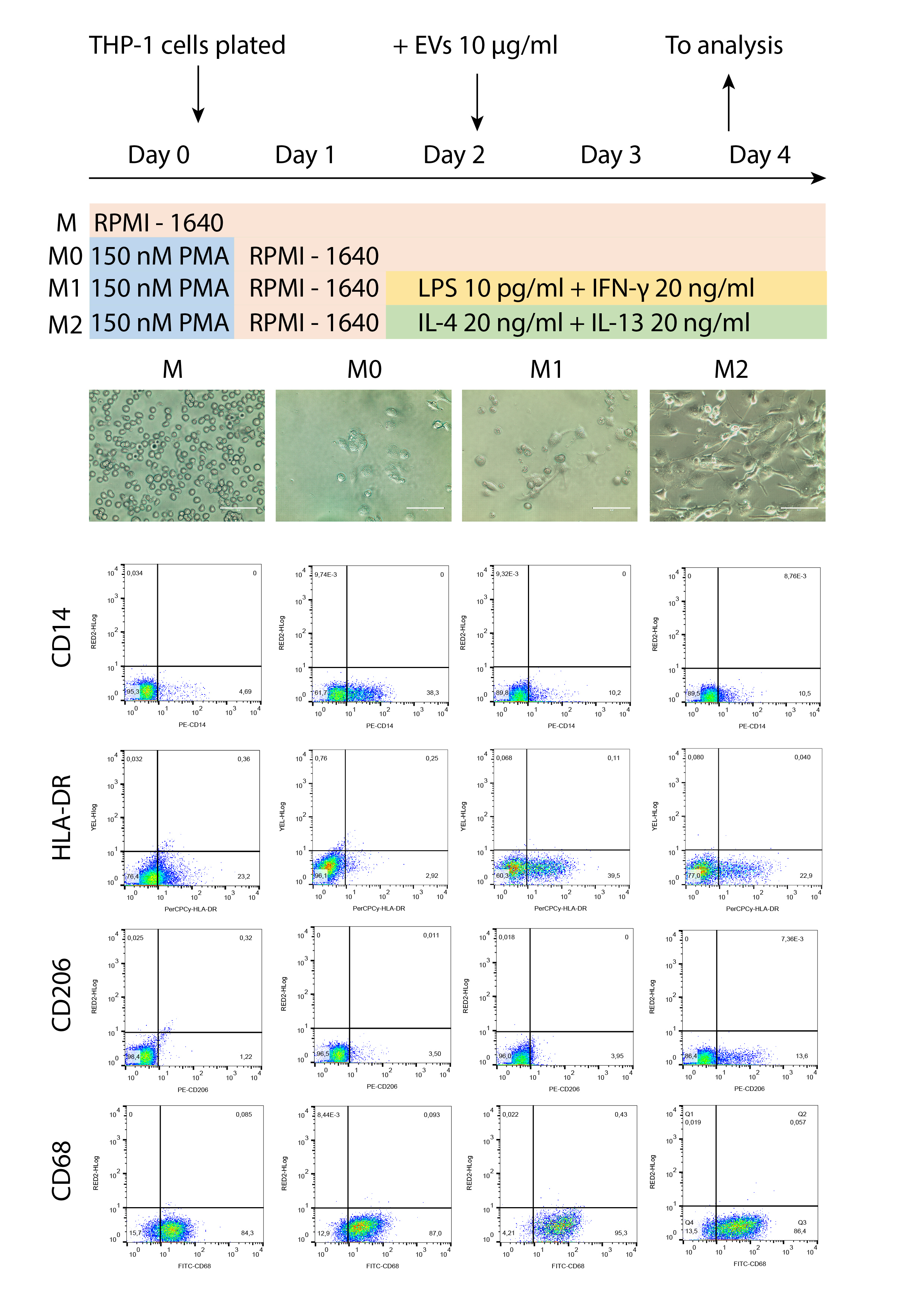

Supplement: Supplementary file 1 — Experimental design of the THP-1 monocyte to macrophage differentiation showing the time points for the addition of EVs and stimulatory molecules. Below the experimental design, representative light microscopy images show morphology of THP-1 monocytes (M), M0 macrophages (M0), M1 macrophages (M1) and M2 macrophages (M2) (n = 4). Scale bar 100 μm. Representative flow cytometry dot plots show CD14, HLA-DR, CD206 and CD68 marker expression at M, M0, M1 and M2 stages. (JPG 1845 kb) [file 12964_2018_229_MOESM1_ESM.jpg]

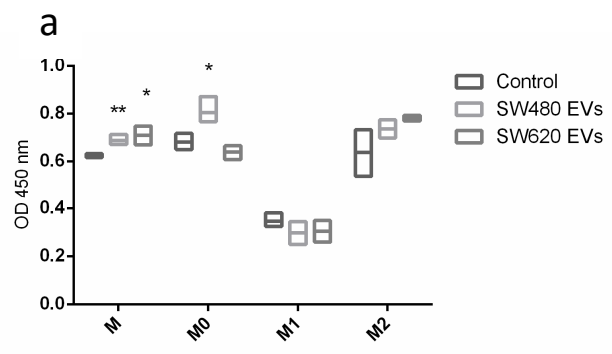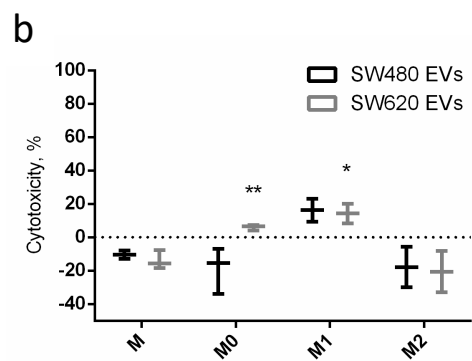

Supplement: Supplementary file 2 — SW480 and SW620-derived EV effect on monocyte (M) and macrophage (M0, M1, M2) viability. a OD values at 450 nm which are in direct proportion of viable cell counts. b SW480 and SW620 EV cytotoxicity on THP-1 monocytes and M0, M1 and M2 macrophages. The graphs represent mean ± SEM (n = 3). Statistical analysis carried out with the t-test. *p ≤ 0.05, **p ≤ 0.01 vs. untreated cell control of the respective monocyte-macrophage cell subset. (PDF 50 kb) [file 12964_2018_229_MOESM2_ESM.pdf]

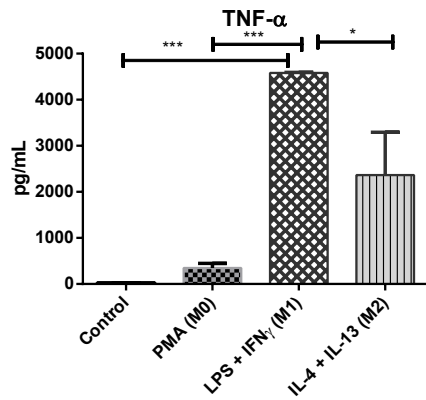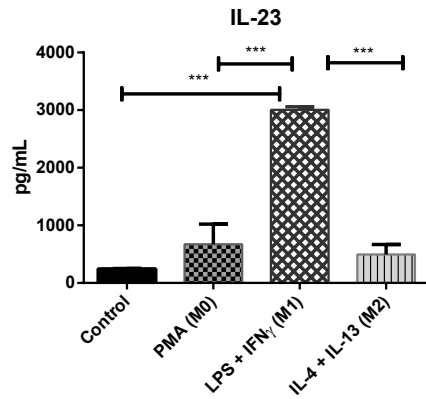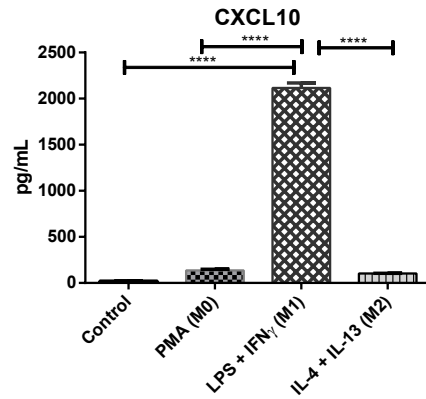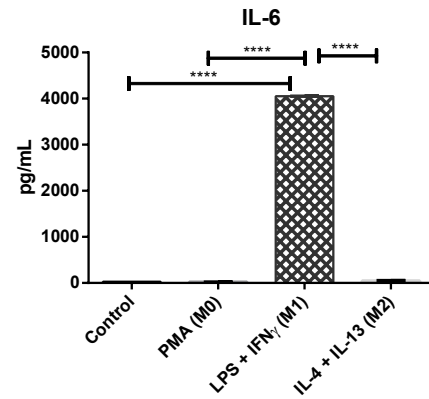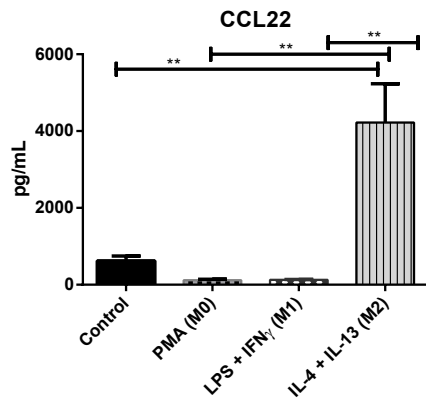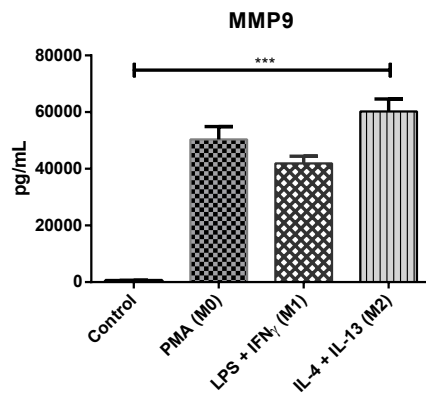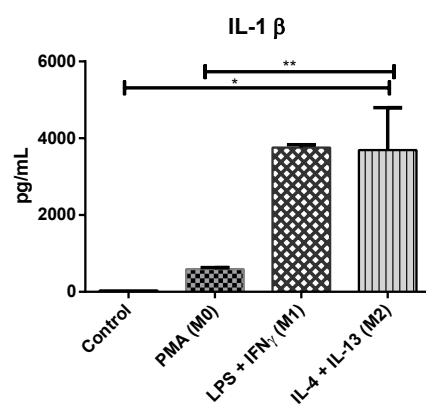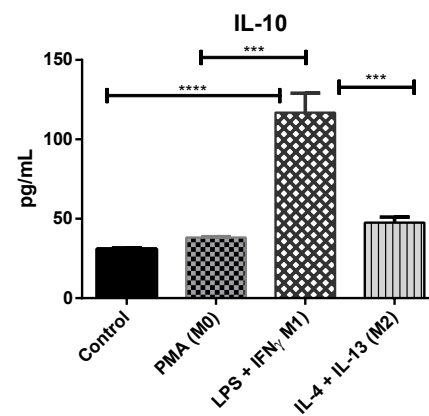

Supplement: Supplementary file 3 — Effect of temperature on the SW480 EV uptake in THP-1 monocytes. Flow cytometry histograms showing Syto RNA Select fluorescence intensities of untreated (left) and Syto RNA Select-labeled SW480 EV-treated THP-1 monocytes following incubation at 4 °C (middle) and 37 °C (right). Histogram markers show the percentage of Syto RNA Select-positive cells. (PDF 53 kb) [file 12964_2018_229_MOESM3_ESM.pdf]

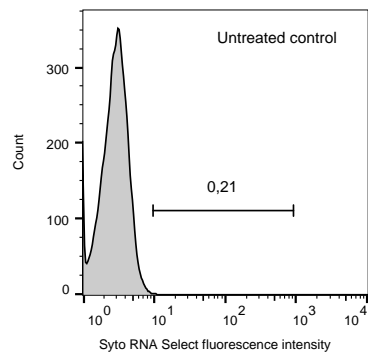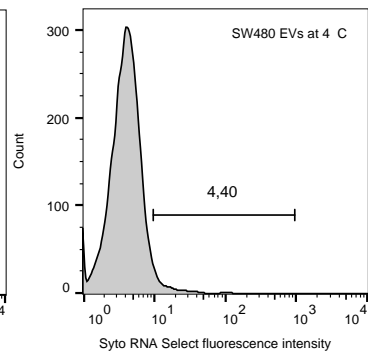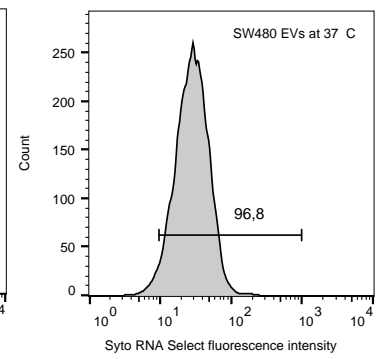

Supplement: Supplementary file 4 — TNFα, IL-23, IL-6, IL-1 β, CXCL10, CCL22, IL-10 and MMP9 secretion profile at different monocyte-macrophage differentiation stages. The graphs represent average biomolecule concentrations SEM (n = 3). Statistical analysis carried out with one-way ANOVA test. *p ≤ 0.05, **p ≤ 0.01, ***p ≤ 0.001 and **** ≤ 0.0001 vs. untreated cell control of the respective monocyte-macrophage cell subset. (PDF 63 kb) [file 12964_2018_229_MOESM4_ESM.pdf]

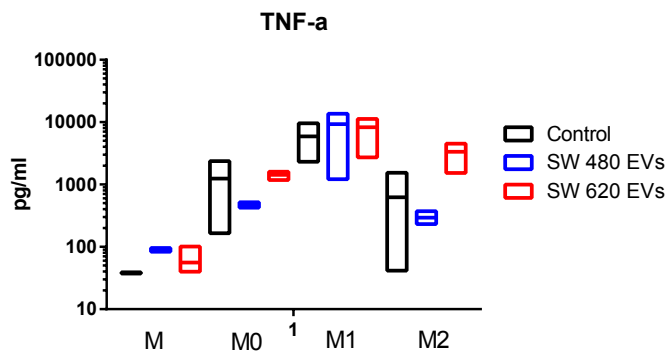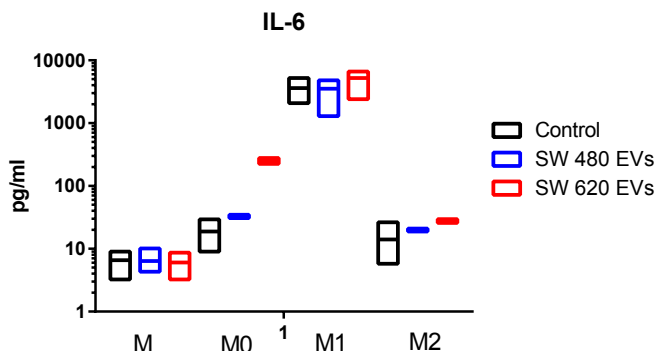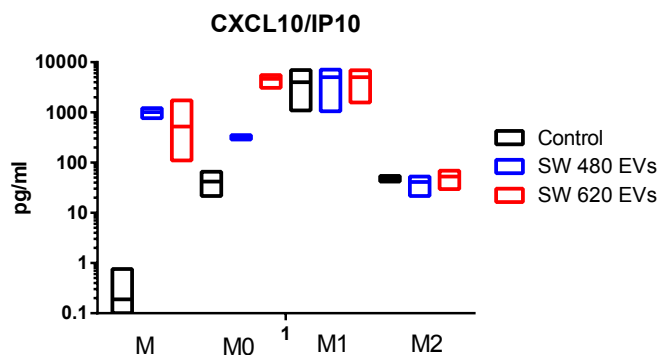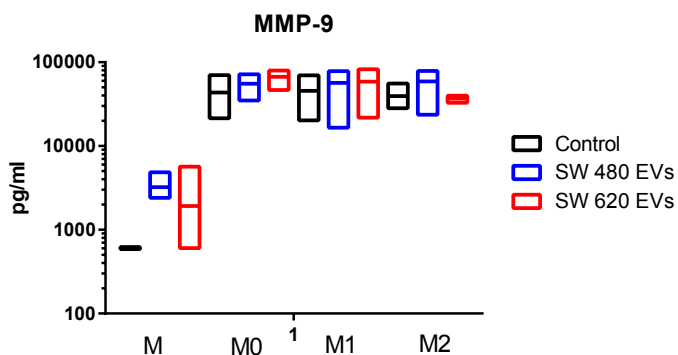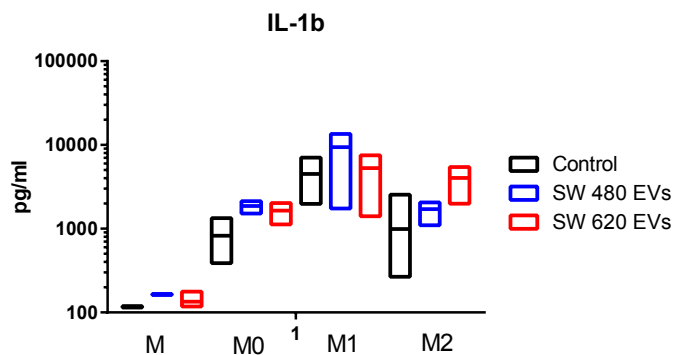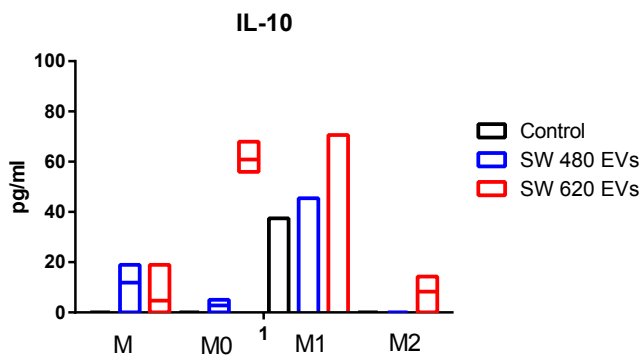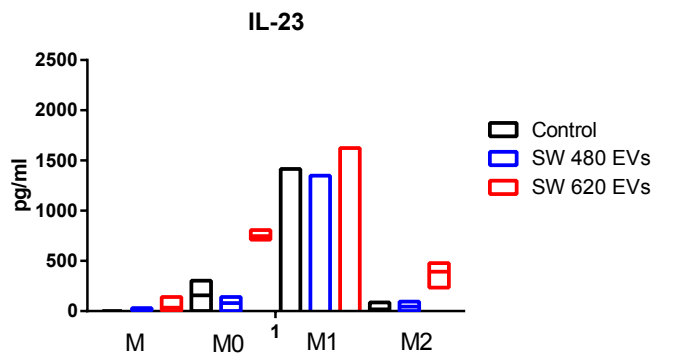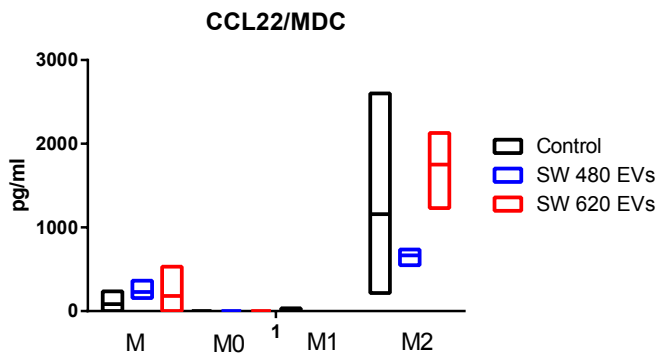

Supplement: Supplementary file 5 — Effect of SW480 and SW620-derived EVs on biomolecule secretion patterns of monocytes and M0, M1 and M2 macrophages. Luminex data analysis showing TNFα, IL-6, CXCL10, IL-23, IL-10, MMP9, IL-1β and CCL22 concentration in cell culture supernatants of monocytes (M) and M0, M1 and M2 macrophages following incubation with SW480 and SW620 EVs or without them (control). The graphs represent mean ± SD (n = 3). (PDF 39 kb) [file 12964_2018_229_MOESM5_ESM.pdf]
